# Blood and Blood Components: From Similarities to Differences

**DOI:** 10.3389/fmed.2018.00084

**Published:** 2018-04-09

**Authors:** Olivier Garraud, Jean-Daniel Tissot

**Affiliations:** ^1^Faculty of Medicine, University of Lyon, Saint-Etienne, France; ^2^Institut National de la Transfusion Sanguine, Paris, France; ^3^Transfusion Interrégionale CRS, Epalinges, Switzerland; ^4^Faculty of Biology and Medicine, University of Lausanne, Lausanne, Switzerland

**Keywords:** transfusion, blood donation, blood processing, blood components, ethics

## Abstract

Blood transfusion is made possible because, in most countries and organizations, altruistic individuals voluntarily, anonymously, and generously donate (without compensation) either whole blood or separated components that are then processed and distributed by professionals, prior to being allocated to recipients in need. Being part of modern medicine, blood transfusion uses so-called standard blood components when relative to cellular fractions and fresh plasma. However, as will be discussed in this paper, strictly speaking, such so-called labile blood components are not completely standard. Furthermore, the prevalent system based on voluntary, non-remunerated blood donation is not yet universal and, despite claims by the World Health Organization that 100% of blood collection will be derived from altruistic donations by 2020 (postponed to 2025), many obstacles may hinder this ambition, especially when relative to the collection of the enormous amount of plasma destined for fractionation into plasma derivative or drugs. Finally, country organizations also vary due to the economy, sociology, politics, and epidemiology. This paper then, discusses the particulars (of which ethical considerations) of blood transfusion diversity and the consequences for donors, patients, and society.

## Introduction

Blood and blood components (BCs) for transfusion chiefly originate from donations made by altruistic individuals. However, although 100% voluntary non-remunerated blood donation (VNRD) is the goal that has been set (by 2020 as publicized by the World Health Organization WHO—later revised to 2025 in some areas) (1, 2), it is far from being achieved at the present time for various reasons linked to contingency (3), and the emergence in low/middle-income countries of modern health-care services requiring more transfusion prescriptions. Blood for therapeutic use comes into two sets: (1) the one consists in labile blood components (LBCs) comprising essentially the cellular components [red blood cells concentrates (RBCCs) and platelet concentrates, PCs as well as part of therapeutic plasma, principally fresh frozen plasma FFP]; and (2) the other one consists in plasma derived or fractionated drugs, and occasionally in FFP obtained from large pools and subjected to stringent pathogen reduction. While the former is principally handled by blood establishments (BEs), many of them overruled by the public sector or Non Governmental, non-for-profit, organizations such as the Red-Cross/Red-Crescent, the latter is largely handled by the plasma fraction industry within the private, for-profit sector.

Labile blood components are usually labeled as “standard blood products,” and thus refer to guidelines such as the Council of Europe’s “Guide to the preparation, use and quality assurance of blood components” (4). However, all LBCs referring to the same label are, in fact, significantly different from each other: they depend on the donor, the process and the storage characteristics which are not consistent, as each consists either in single donor originating units or small pools (5 in mean, and >12 by all means); minipools apply to PCs and therapeutic plasma. Indeed, as each LBC reflects both the genetic and non-genetic-based characteristics of the donors (referred to as “storage lesions”), they all differ from one another despite considerable efforts made to minimize deviations in their process.

This paper will briefly outline the most visible community forms of and differences in LBCs, and the way in which differences can affect outcomes in: (1) recipients; (2) health policies and economics (as essential additional safety procedures can result in even more complex disparities between LBC inventories). The circumstances of blood collection and how ethics are concerned when blood supply does not meet demand or when marketing creates new markets will also be discussed.

## As the Essential Determinant in Transfusion, Blood Represents a Common Matter for Humans

Every human depends on nearly normal blood functions to survive. Moderate alterations may result in sub-physiological functioning, while more severe defects can be corrected by either drugs or blood derivatives (when available), or both, pending anticipated gene correction. Blood characterizes species and cannot be safely exchanged between species, even close ones such as humans and apes. Human blood transfusion can nevertheless be processed within the human species provided major compatibility rules are respected. This property first allowed blood transfusion to be performed in emergency situations, and later, with technological advances, to be applied almost routinely, despite the fact that “routine” is a word that never applies to blood transfusion from either the donor’s or the recipient’s perspective (5, 6).

Donated blood is humanity (7, 8) and ideally, VNRD blood is “pure” humanity, yet replacement donation shares the values of assistance and cannot be considered non-altruistic, even if a certain degree of social pressure cannot be ruled out (3). Maybe the lowest common denominator is the root symbol of blood, its sacred characteristic, whatever sacred may mean to people, ranging from religious to atheistic sentiments (9). Thanks to initiatives like Blood Donor Day on the 14th of June, it can be acknowledged on the highest scale that many people worldwide share the idealistic human view of blood donation for transfusion purposes (10). Ideally, this standpoint should make sure that such a process expands and infuses in areas where VNRD is not yet achievable. However, the situation is far from universal or exemplary. Three taints can be outlined as follows: (1) as the demand for BCs expands in many countries, BEs start applying marketing methods to attain prospective new blood donors, potentially altering the voluntary aspect of donation, or the benevolence (or the absence of profit) (11). The same holds true for plasma collectors within the industry (12–16). (2) The benefits of blood have been distorted to serve non-life-threatening medical conditions, and BCs have entered the for-profit market and business where blood is being increasingly used in welfare clinics for doping or cosmetic applications (17, 18). This merchandized blood may, therefore, no longer be available for therapeutic and medical indications, potentially worsening the shortage problem and ultimately leading BEs to use enhanced marketing tools and enter a vicious circle. (3) Blood and BCs for research now represent an expanding market, they can either be “conventional” as BEs trade left-overs from test tubes or residues from blood processing, or BCs purposefully processed, i.e., BEs collect blood from VNRDs not eligible for the therapeutic pathway or presenting characteristics required for specific research. In those cases, VNRDs are usually informed that all or part of their donation will be used for research programmes and they may object to this use. Moreover, some donors sell blood for specific research programmes, with the blood generally being collected outside of BEs; this is possible in some countries but is strictly forbidden in others. Research ethics committees would prevent these possibilities.

## Similarities of Human Blood

As has already been outlined, one common platform for humans considering blood is the general anthropological consideration of this “fluid” which is both material (the blood tissue) and spiritual or the like (altruism, attraction/repulsion, life and death, genetics and lineages, war and peace, and so forth) (9). Regarding its materiality, human blood is unique to the species, and there is by no means any significant difference between ethnic groups, which refutes previous racist theories on the purity of blood. There are indeed certain variations that vary in their frequency in ethnic groups as will be presented in the next section of this paper, but none of them prevent donated blood from being issued to any other human, apart from major (ABH) blood (in fact, tissue) antigen group compatibility. These fundamental characteristics allow the principle of blood transfusion and, moreover, of universal blood transfusion, once the major restriction of the ABH system is taken care of.

## Diversities in Human Blood and BCs for Transfusion

In contrast to what has been presented above, no siblings have identical blood. Indeed, even monozygotic twins display genetic and epigenetic differences. Whereas the Mendelian distribution of HLA antigens is transmitted in blocks (haplotypes), HLA diversity between humans ranges from between 10^6^ and 10^7^ (19). HLA polymorphism limits, to some extent, platelet and leukocyte availability and may also create complications such as immunization (20). Platelet antigens add diversity by bringing more than 30 additional groups often with two alleles each (about 5 matter essentially in transfusion) (21, 22). The diversity of erythrocyte antigenic alleles is not only huge (by the thousands) but is also non-haplotypic, offering a tremendous assortment of individual cell markers which is fascinating if we consider the uniqueness of each human, but limits the possibility of matching blood groups for transfusion (23). In fact, half a dozen such antigens are considered routine, allowing for wide-scale transfusion. It should be noted that not all individuals are equal and some individuals can be easily transfused (the vast majority) while others with rare blood groups cannot, requiring special programs with technical, ethical, and organizational problems. Similarly, there is diversity in people’s propensity to become alloimmunized after blood transfusion, because HLA loci make people good or bad responders to RBC, platelet, or leukocyte antigens (a common law in innate and adaptive immune responses that varies in individuals) (24–26).

With respect to LBCs, despite being called “standard,” and their largely similar appearance category by category, they are not, in fact, all the same. They are defined as standard because they fall within a range of maximum and minimum levels of either desired and undesired constituents, and only a few parameters are evaluated among the thousands of variables that make individuals’ blood differ from another (4). Furthermore, for each category of LBC, besides the genetic variations between donors that transfer characteristics to the donated component (gender, tissue and HLA genotype, erythrocyte antigen genotype, platelet antigen genotype, protein variants, etc.), there are additional variation parameters that depend on the donors: the time of donation (morning, afternoon, after a meal or fasting, drug and dietary supplement intake, menstrual cycle for non-menopaused women, diet, hygiene habits, and many other parameters) (27–29). All of these parameters may influence the final characteristics of the LBC, though there is no evidence as yet of the effect apart from the presence of soluble antibodies against blood cell antigens (iso-anti-A or B; anti-HLA in females who have been pregnant). BEs extend the variety since the addition and diversification of devices, machines and automats multiply heterogeneity (Table 1 aims to illustrate this). Finally, shelf-life duration multiplies LBC delivery diversity by 41 for RBCCs (in theory: 42 minus 1 day, as 1 day is needed to obtain all the parameters allowing the labeling and issue of the RBCC) (30) and 4 (5 minus 1 day) for PCs. It has further been noticed near two decades ago that RBCs do not recirculate the same depending on storage conditions of the component (one ¼ of the infused RBCs even never recirculate) (31).

**Table 1:** Parameters having proven or theoretical influence on the quality of the processed blood component (BC).

Main categories	Main items adding diversity	Level of diversity
Donor dependent parameters (genetically controlled)	Sex/gender Immunogenetic characteristics (blood groups) Natural iso-antibodies …	Two By the thousands (millions if applied also to HLA antigens) Variable

Donor dependent parameters (only partly genetically controlled)	Immunization status Nutrition, metabolism Hygiene and intoxications (therapeutic and recreational drugs, supplements, alcohol, tobacco) Meal; or fast Nycthemeral cycle Genital cycle and periods Outside temperature condition …	Hundreds of influential parameters

Donor independent parameters (BC processing)	Shipping time and temperature Needles, plastics and bags, rotators, automats for collection and intermediate storage Devices for cell separation Working temperature Additives (anticoagulant, solutions, pathogen inactivation, etc.) Filtration steps (meshes, temperature, timing, etc.) Pooling steps Preservation conditions Physical interactions in shelf-life conditions (stacking, shocks, thermic differences, shipping, etc.)	Variable Dozens influential parameters

Patient (recipient, beneficiary) dependent parameters	Blood group Immunization status Matching conditions	By the thousands

Each parameter being independent from the preceding one, diversity is created by the multiplication as opposed to the addition of all. The final diversity goes by the million or more. Not all parameters are equally influential but it clearly appears from the table that one given BC collected by one individual, despite being “standardized” to a norm, is unlikely to be “standard.”

What then is the clinical relevance of such diversity? The case of storage lesions has been extensively studied (32–34), having basically identified two sets of lesions: reversible and irreversible lesions (35). Conflicting data have been obtained from both experimental and clinical data, sparking disputes over the rationale for using fresh as opposed to old blood (36–38). In simple terms, it appears that neither fresh nor old blood is clearly defined, nor is the readout (patient outcome) providing conclusive information, and relevant clinical trials are still needed to resolve this issue (39–41). This issue is of utmost importance as it may revoke the current blood-banking paradigm (meaning that conflicts of interest when blood banks happen to be research principal investigators are non-negligible).

## Disparities in Public Health Policies

Labile blood components are not considered univocal in various countries and systems, though they are not considered to be drugs according to Directive 2002/98/EC (42), in contrast to plasma fractions or derivatives [Directive 2001/83/EC (43)], or industrially prepared and solvent-detergent secured therapeutic plasma originating from large pools. Some national regulations do, however, consider labile LBCs to be drugs, as is the case in Switzerland; and WHO recently (2015) added blood and blood derivatives to its list of essential medicines (44). Semantics between medicines and drugs are imprecise, however, and this decision has displeased a number of blood donor associations who are batting against confusion with LBCs and plasma derivatives (45). Furthermore, the logistics by which certified LBCs can be delivered to patients varies considerably between countries: according to the European directive, there are basically two main actors: BEs which collect, process, test, and distribute BCs, and Hospital Blood Banks or HBBs which acquire LBCs from BEs, build up an inventory and deliver selected LBCs to patients upon *ad hoc* immunohaematological matching (4). On some occasions, BE and HBB functions can be held by the same entity (as, e.g., in France).

In addition, Council of Europe directorate EDQM requests that BEs apply a minimum platform for blood testing, with additional options for BEs willing to raise the LBC safety level with, for example, the implementation of nucleic acid testing (NAT) (4). Some BEs perform NAT on each donation, even on frequent donors’ donations, while others restrict it to new donations. NAT is applied to both pools and individuals, and some BEs that used NAT have reverted back to non-NAT because of its relatively low (and debatable) cost-effectiveness (46).

In short, there is significant heterogeneity in stances on the availability of LBCs to patients through organizations and countries’ public health policies. Consistent efforts have been made by large bodies such as EDQM, the American Blood Bank Association or the International Society for Blood Transfusion ISBT to define safety and quality parameters that should apply to each issued LBC; however, this is still a range above and beyond which the LBC should not conform to standards; if testing is individual for most infectious markers (and even some are tested in pools), hematological markers are frequently tested by sampling to define quality (4). This is the reason why we call attention here on the false homogeneity of LBCs despite they go by the noun of “standard.” This is in sharp contrast to the actual standardization of plasma derivatives obtained from 100 of 1000s of collections and subjected to an industrial processing (47).

## Disparities within Ethical Considerations

Ethics are largely cultural and it is difficult to establish a platform that fits all situations worldwide. Nevertheless, as early as 1975, WHO declared that sustainable efforts should be made by all countries to collect blood from VNRDs. WHO later set the objective of 100% VNRD by 2020 (the Melbourne Declaration, 2009) (1). The EDQM has also declared itself in favor of a generalized objective of 100% VNRD where possible (4). The Oviedo Convention (1997), set up under the auspices of the Council of Europe, has established standards for ethics in the field of human rights and dignity, with regard to the use of tissues of human origin (including blood) (48). Nonetheless, many states have been late in signing this convention and others have not yet ratified it. WHO, considering that many countries could not achieve the 2020 VNRD objective, postponed it to 2025 for certain Middle-Eastern countries. State WHO and ISBT in its revised code of Ethics, paid and compensated donations should no longer be acceptable; replacement donations remain questionable (49). The issue of paid versus unpaid plasma collection has been challenged by advocates for the plasma fractionation industry (13, 14, 16) (the case of paid cell donations is still existing in certain countries including Europe but apparently on the decline worldwide). It is worth noting that a very interesting study was published in 2012 questioning the ban on financial contracts with blood donors in Africa, with the declared objective to reduce the infectious burden on “donated” blood (50). This last example questions the universality of ethical values of blood donation when coming to strengthening safety in LBC beneficiaries, opening an additional ethical dilemma. Next, when this comes to plasma for fractionation, there have been requests that collection and fractionation are more equally dispatched in countries that prescribe most to do justice to patients in need in case there is shortage and to alleviate the burden of (paid) collection in countries hosting the majority of plasma collectors such as the USA (51).

We offer an opinion here: as opposed to normative ethics (the Oviedo Convention, the ISBT Code of Ethics), reflective ethics (anthropological and philosophical) are preferred in order to move forward with the safe use (for donors, recipients, stakeholders and society members, i.e. people, civilians and tax payers) of substances of human origin (41). In the meantime, there is clear evidence that ethics are extremely cultural and embedded with acknowledged or hidden religious sentiments with the populations concerned.

In short, it should be noticed that blood within the transfusion process offers an additional paradox: while the LBCs are relatively inhomogeneous relative to active constituents though relatively homogenous relative to the so-called ethicality of their collection, this is the other way around for plasma derivatives that are remarkably homogenous and standardized relative to active constituents but may be obtained both within the profit and the non-for-profit sectors. The latter case has been recently (2016) the subject of important debated in France and Switzerland and journalists reported both in the paper and the TV press on some potential malpractice with respect to donors’ own safety and vulnerability.

## Concluding Remarks

The transfusion process is at the crossroads of a multitude of paradoxes (42). It is one of the only medicines that rely on material outside the control of the pharmaceutical industry if we exclude plasma for fractionation. It professes to be precision medicine, yet the issued LBC comprises ±20% (or more) of the expected therapeutic constituent. It professes to be personalized medicine, but blood groups between donors and recipients match for the vast minority. It is prescribed for the most fragile patients, yet practitioners prove to have very limited knowledge on it (according to recent surveys, they usually know which is the target of a biosimilar drug or a therapeutic monoclonal antibody, but only largely relative to LBCs) (43). We believe it is important to advocate for prescribing doctors to receive further training on LBCs and transfusion medicine–setting apart the case of standardized plasma derivative or drugs—education and training are essential to understanding and respecting blood donors and donated LBCs, to making wise choices, and to the optimal management of patients’ blood and donated LBCs, within the limitations of prevailing uncertainty (Garraud et al., in preparation). Being knowledgeable in the face of uncertainty would prevent a comparison of apples and oranges in published works, even in high-quality journals. It would be practical for prescribing doctors to forward their everyday questions to blood transfusion specialists; this would certainly be useful in establishing the most appropriate training programmes.

## Author Contributions

Both authors contributed equally to this manuscript and its revision.

## Conflict of Interest Statement

The authors declare that the research was conducted in the absence of any commercial or financial relationships that could be construed as a potential conflict of interest. The reviewer PT and the handling Editor declared their shared affiliation.
